# The plant endoplasmic reticulum UPRome: A repository and pathway browser for genes involved in signaling networks linked to the endoplasmic reticulum

**DOI:** 10.1002/pld3.431

**Published:** 2022-07-20

**Authors:** Venura Herath, Kaylee Connolly, Anna Roach, Ashish Ausekar, Tracy Persky, Jeanmarie Verchot

**Affiliations:** ^1^ Department of Plant Pathology & Microbiology Texas A&M University College Station Texas USA; ^2^ Department of Agriculture Biology, Faculty of Agriculture University of Peridaniya Peradeniya Sri Lanka; ^3^ Division of Information Technology Texas A&M University College Station Texas USA

**Keywords:** curated database, plant protein interaction maps, plant signal transduction, unfolded protein response

## Abstract

The endoplasmic reticulum (ER) houses sensors that respond to environmental stress and underly plants' adaptative responses. These sensors transduce signals that lead to changes in nuclear gene expression. The ER to nuclear signaling pathways are primarily attributed to the unfolded protein response (UPR) and are also integrated with a wide range of development, hormone, immune, and stress signaling pathways. Understanding the role of the UPR in signaling network mechanisms that associate with particular phenotypes is crucially important. While UPR‐associated genes are the subject of ongoing investigations in a few model plant systems, most remain poorly annotated, hindering the identification of candidates across plant species. This open‐source curated database provides a centralized resource of peer reviewed knowledge of ER to nuclear signaling pathways for the plant community. We provide a UPRome interactive viewer for users to navigate through the pathways and to access annotated information. The plant ER UPRome website is located at http://uprome.tamu.edu. We welcome contributions from the researchers studying the ER UPR to incorporate additional genes into the database through the “contact us” page.

## 1 INTRODUCTION

The plant endoplasmic reticulum (ER) is a dynamic network of membranous tubules and sheets that is continuous with the nuclear envelope and extends across plasmodesmata into neighboring cells. The ER is best known for its role in translation, protein folding and maturation, and lipid synthesis. It is the major production organelle for soluble and membrane‐bound proteins that are destined for various organelles including the golgi, vacuole, and plasma membrane, as well as the apoplast (Brandizzi et al., 2002; Ellgaard et al., 2016; Ruberti et al., 2015; Schwarz & Blower, 2016). The ER quality control (ERQC) system supervises the multiple process steps in protein maturation and secretion. It is responsible for resolving stress conditions that can interfere with delivering mature proteins to their destinations. The ERQC is important for plant growth, metabolism, and adaptation to environmental assaults (Ruggiano et al., 2014; Sun et al., 2021; Tateda et al., 2008; Verchot, 2012). Understanding the integration of stress signals and how conditions can impair ER function is critical to understanding plant development and immunity.

Across eukaryotes, the unfolded protein response (UPR) consists of three or more major branches led by ER resident stress sensors. In mammals one branch is led by the membrane tethered transcription factor 6 (ATF6). Another branch is led by the inositol requiring enzyme 1 (IRE1, a and b isoform), which is a type 1 transmembrane protein kinase/endoribonuclease. The third branch is led by four kinases that mediate phosphorylation of eukaryotic translation initiation factor 2a (elF2a): (a) GCN2 (General Control Non‐repressible 2); (b) PERK (RNA dependent Protein Kinase like ER kinase, also known as EIF2AK4); (c) HRI (Heme Regulated Inhibitor); and (d) PKR (Protein Kinase R) (Hollien, 2013; Verchot & Pajerowska‐Mukhtar, 2021). In plants, the ER‐resident sensors include three homologs of IRE1 named IRE1a, IRE1b, and IRE1c; two transcription factors bZIP17 and bZIP28 that resemble the mammalian ATF6; and a single GCN2 orthologue that phosphorylateselF2α (Verchot & Pajerowska‐Mukhtar, 2021). The IRE1s have an endonucleolytic activity that splices an unconventional intron of the mRNA encoding the transcription factor XBP1 in mammals and bZIP60 in plants. The truncated XBP1 and bZIP60 transcription factors function to regulate ER stress‐responsive genes. Arabidopsis has additional ER to nuclear signaling pathways led by the membrane‐bound AtbZIP49 and AtNAC089 transcription factors, or the transcription co‐factor AtBAG7 (Li et al., 2017; Nawkar et al., 2018; Tajima et al., 2008; Yang et al., 2014). While the stress trigger and gene targets for AtbZIP49 are unknown, these other factors contribute to hormone signaling, ROS homeostasis, and disease resistance (Bao & Howell, 2017; Nagashima et al., 2014; Nawkar et al., 2018; Verchot & Pajerowska‐Mukhtar, 2021; Wang et al., 2014).

Angiosperms have undergone intensive gene expansion, and polyploids have undergone more than one duplication event leading to an overall expansion of gene families (Lespinet et al., 2002). Combined with the whole genome expansion are segmental and tandem duplications and the broad need for expanded stress adaptation. This makes the number of gene family members in angiosperms larger than in *Drosophila* and *Homo sapiens* and achieves more complex gene regulatory networks. For example, the bZIP family is categorized as Group A through Group M plus Group S and the Arabidopsis groups B and K are described as ER stress‐affiliated factors. Arabidopsis has four group B/K members, *bZIP60*, *AtbZIP17*, *AtbZIP28*, and *AtbZIP49*. The group B/K genes include 9 members in *brassica napus*, 5 in cucumber, 14 in maize, 6 in rice, 7 in potato, and 8 in wheat (Agarwal et al., 2019; Baloglu et al., 2014; Herath & Verchot, 2020; Ji et al., 2009; Wang et al., 2011; Wei et al., 2012; Zhou et al., 2017).

Until now, annotated genes involved in ER‐to‐nuclear signaling across angiosperms can be difficult to access. Gathering such information is essential to gain insights into molecular mechanisms for stress regulation and their contributions to useful agronomic traits. Such information can aid researchers interested in signal transduction networks either for fundamental studies, genetic engineering, or crop improvement. Published datasets of gene expression data make it possible to link environmental stimuli to genome‐wide analysis studies of plant gene families, it remains possible that gene family members within the current phylogenies may contribute to new functional categories across a range of plant species because of their expansion.

We created a user‐friendly database that integrates publicly available resources to meet the ongoing challenge for researchers to address the abundant and complex biological information that exists across plant species. The UPRome annotation database is a resource for comparative pathway analysis, and multi‐omics datasets include manually curated pathway information built on peer‐reviewed literature and datasets. To better understand ER to nuclear signaling, we created this database to provide the most current information on these signaling networks across plant species. The database contains orthologues across distant angiosperms and links to various plant genome databases.

The database includes UPR relevant factors that influence protein maturation processes, and parallel ER to nuclear signaling pathways. The website consists of a graphic interface that provides direct links to genes and proteins across plant species. The current database includes genes from Arabidopsis, maize, potato, rice, soybean, and tomato, which represent the most studied plant models for UPR and major food crops. The interactive viewer allows users to click within the image to view external identifiers for these plant species. The external identifiers include hotlinks to online data resources such as relevant plant omics databases, UniProt, NCBI RefSeq, NCBI GENE, EnsemblPlants, and ProteomicsDB. The goal of the database is to support basic research, analysis of ER to nuclear signaling pathways, enable genome analysis and modeling, and support systems biology and education.

## 2 MATERIALS AND METHODS

### 2.1 Data collection

We began with a list of core ER‐associated genes related to the UPR in Arabidopsis that are experimentally validated in peer‐reviewed literature downloaded from PubMed and Google Scholar. We obtained the gene names, gene IDs, and DOI links from the primary literature. Using the AmiGO 2 Gene Ontology browser, we found the total number of genes and accessions using enrichment terms for cell compartments and biological processes: ER (GO:0005788), Golgi (GO:0005829), nucleoplasm (GO:0005654), and cytosol (GO:0005829), ER UPR (GO: 0030968), cellular response to unfolded protein (GO:0034620), and response to unfolded protein (GO:0006986). We built an internal database (Microsoft Excel ver 2204) focusing on key model plants: Arabidopsis, *Glycine max* (soybean), *Oryza sativa* (rice), *Solanum lycopersicum* (tomato), *Solanum tuberosum* (potato), and *Zea mays* (maize). The total number of gene accessions differed significantly from the total number of genes for each plant species in each GO category. This indicated that there were likely duplicated gene entries, multiple accessions attributed to a gene, and possibly deleted entries for misidentified genes. Therefore, we built an internal database with protein names, Gene identifiers (IDs) provided in all the appropriate plant genome databases, EMBL (UniProt, EnsemblPlants, and Proteomics DB), and NCBI (RefSeq, Gene ID, mRNA sequence). This database contained IDs and hotlinks which were manually confirmed as the core accessions across databases that consistently identify genes and orthologues. For questionable entries, the sequences were downloaded to Geneious Prime, and searched by BLAST obtain Gene IDs for comparison. Then sequence alignments were performed using Geneious Prime. Data were manually verified before building into the public database. The last access date for the genome databases is June 3, 2022.

The interactive viewer was built using an illustration constructed in BioRender, an online tool for science figures. Figures for this manuscript were constructed using Adobe Illustrator.

### 2.2 Database construction

The plant UPRome website at http://uprome.tamu.edu was created using Microsoft's ASP.NET web framework version 4.8 and the C# language. The website also uses a Scalar Vector Graphics (SVG) responsive image to display the UPRome image with clickable links for proteins to display external identifiers and external database hotlinks. The user interface uses Bootstrap version 5.0 and responsive design to be functional across all devices, from PC to mobile devices. All website data is hosted on a Microsoft SQL Server database. The database contains tables of plant species, genes, proteins, and links to external identifiers. Over time, more species and proteins will be added. The original data is managed in relational tables in Microsoft Excel. External identifiers include Protein IDs, Plant Species IDs, External Database IDs, and Gene Model IDs and link to the parent Plant Species and Protein Name in the hierarchy. The External Identifiers are associated with hyperlinks to external databases.

## 3 RESULTS

### 3.1 The user Interface

**FIGURE 1:**
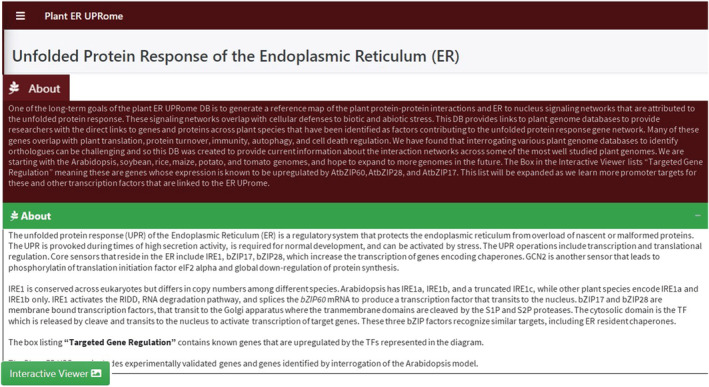
Plant endoplasmic reticulum (ER) UPRome. The panels of information that the readers will see on the front page of the database.

There are three major items on the homepage of the plant ER UPRome database (Figure 1). A maroon‐colored banner explains the project goal to provide genetic information from various annotated plant genome databases to present a reference map of signaling pathways that stretch from the ER to the nucleus. ER stress sensors reside at the top of these signaling networks. The current collection of factors will expand as researchers discover additional stress sensors and regulatory proteins associated with the ER. The current Plant ER UPRome includes factors involved in protein translation, protein turnover, protein folding, immunity, autophagy, and cell death regulation. A green banner explains that the core function of the UPR builds on the availability of protein chaperones for the folding of nascent proteins, the integrity of disulfide bridges, and the quality of protein glycosylation under normal cellular conditions and conditions of cellular stress. This green banner takes the users to an interactive viewer hosted on another page. The database provides gene ontology terms for cellular compartments, biological processes, and keywords for PubMed references. A schematic diagram represents the UPR signaling pathways and a list of genes that are regulated by the bZIP transcription factors (in a box labeled “Targeted Gene Regulation Factors”). The diagram presents ER‐resident factors related to growth and ER stress management, fatty acid synthesis, oxidative stress responses, cold and heat tolerance, and cell death regulation which relate to various environmental challenges. Gene expansion has occurred across angiosperms from which is inferred the likelihood that factors engaged in ER to nuclear signaling have also expanded, and functions may exist in some plant species that do not occur in Arabidopsis. The front page also presents access to an interactive viewer that takes the users to a separate web page where they can explore genetic information across Arabidopsis, soybean, rice, maize, potato, and tomato. Key cellular compartments and biological processes are listed and will be updated routinely.

### 3.2 Data retrieval across plant genomes

**FIGURE 2:**
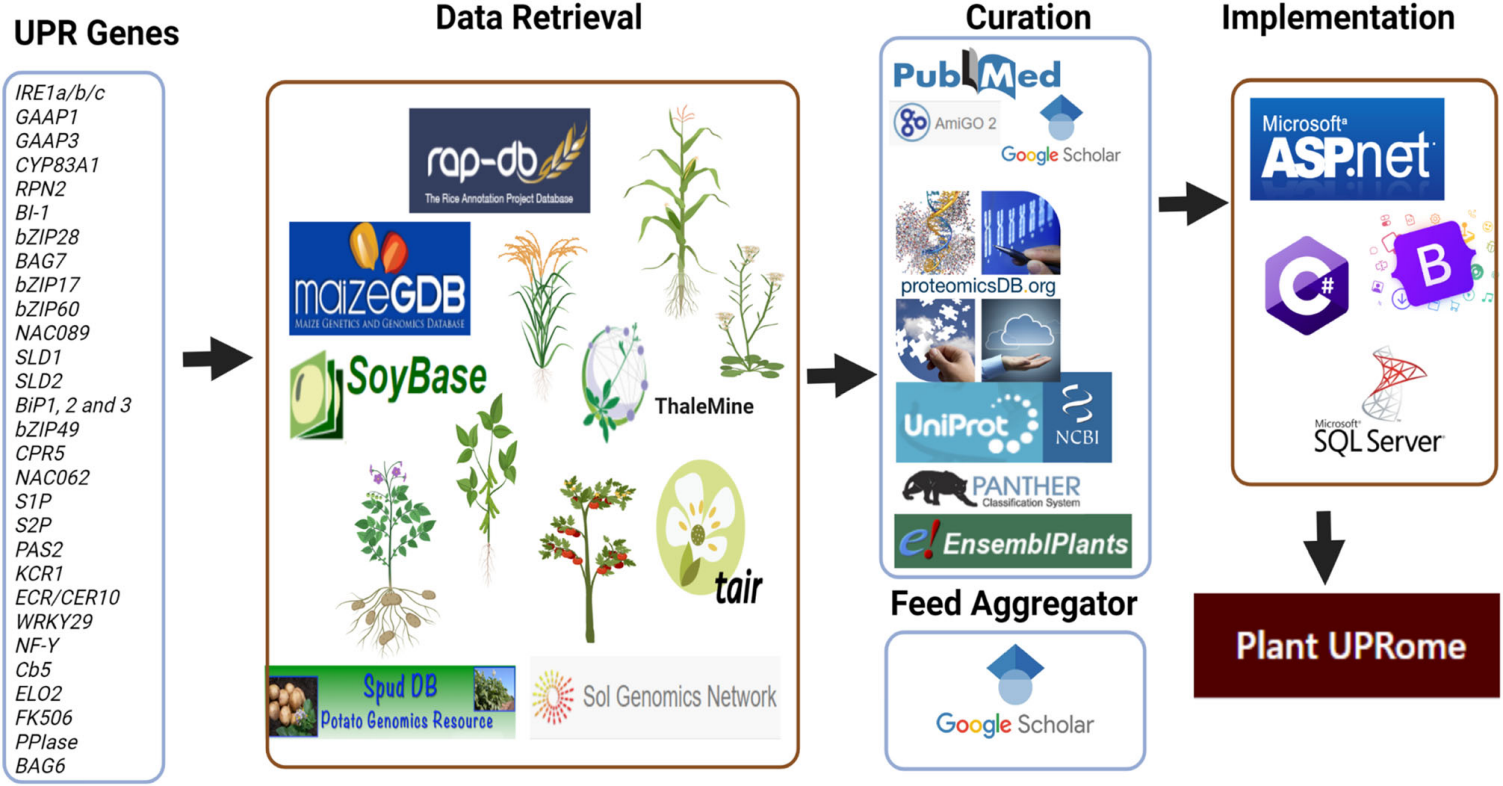
Plant UPRome workflow. The database was constructed based on the reported unfolded protein response (UPR) associated genes in model plant species esp. *Arabidopsis thaliana* . Homology based gene retrieval was carried out against major plant genome databases as indicated in the second panel. The retrieved genes were manually curated after careful inspection of their sequences, domains, and motifs as shown in the top third panel. We also incorporated a feed aggregator so the users can obtain up to date references related to the six plant species included in our database. We used Microsoft ASP.net, C#, Bootstrapper, and Microsoft SQL Server for the implementation of the Plant UPRome.

We retrieved all possible genes and proteins (a catalog of 1209 entries) using Arabidopsis, soybean, rice, maize, potato, and tomato genome databases: TAIR, Thalemine, Maize GDB, Rice Genome Annotation Project (RAP), SpudDB, Sol Genomics Network, and Soybase (Figure 2). Gene IDs and their functions were cross‐referenced between genome databases to validate orthology across genome databases. Then BLAST searches were carried out using Proteomics DB, EnsemblPlants, Panther, UniProt, and NCBI. The BLAST outputs were used to select candidates with *e*‐values between .0 and 1e−15 to include in the ER UPRome database. Then orthologous proteins were validated by manually checking linear arrangements of conserved domains and motifs in UniProt. Table 1 presents the distribution of genes belonging to Plant ER UPRome across six species. Arabidopsis, soybean, tomato, and potato have 48, 53, 44, and 37 genes, while rice and maize have 41 and 44 genes, respectively.

**TABLE 1:** Genes in the plant ER UPRome database

Species	Genes
*Arabidopsis thaliana*	48
*Glycine max*	53
*Oryza sativa*	41
*Solanum lycopersicum*	44
*Solanum tuberosum*	37
*Zea mays*	44

Literature searches were performed using PubMed and Google Scholar to identify publications that investigated various gene functions by knockout mutations, knockdown mutations, or overexpression experiments. Then we created an easily accessible list of publications by generating an RSS feed aggregator linked to Google Scholar using the keyword “unfolded protein response” plus the plant species (Figure 2).

### 3.3 Interactive diagram

**FIGURE 3:**
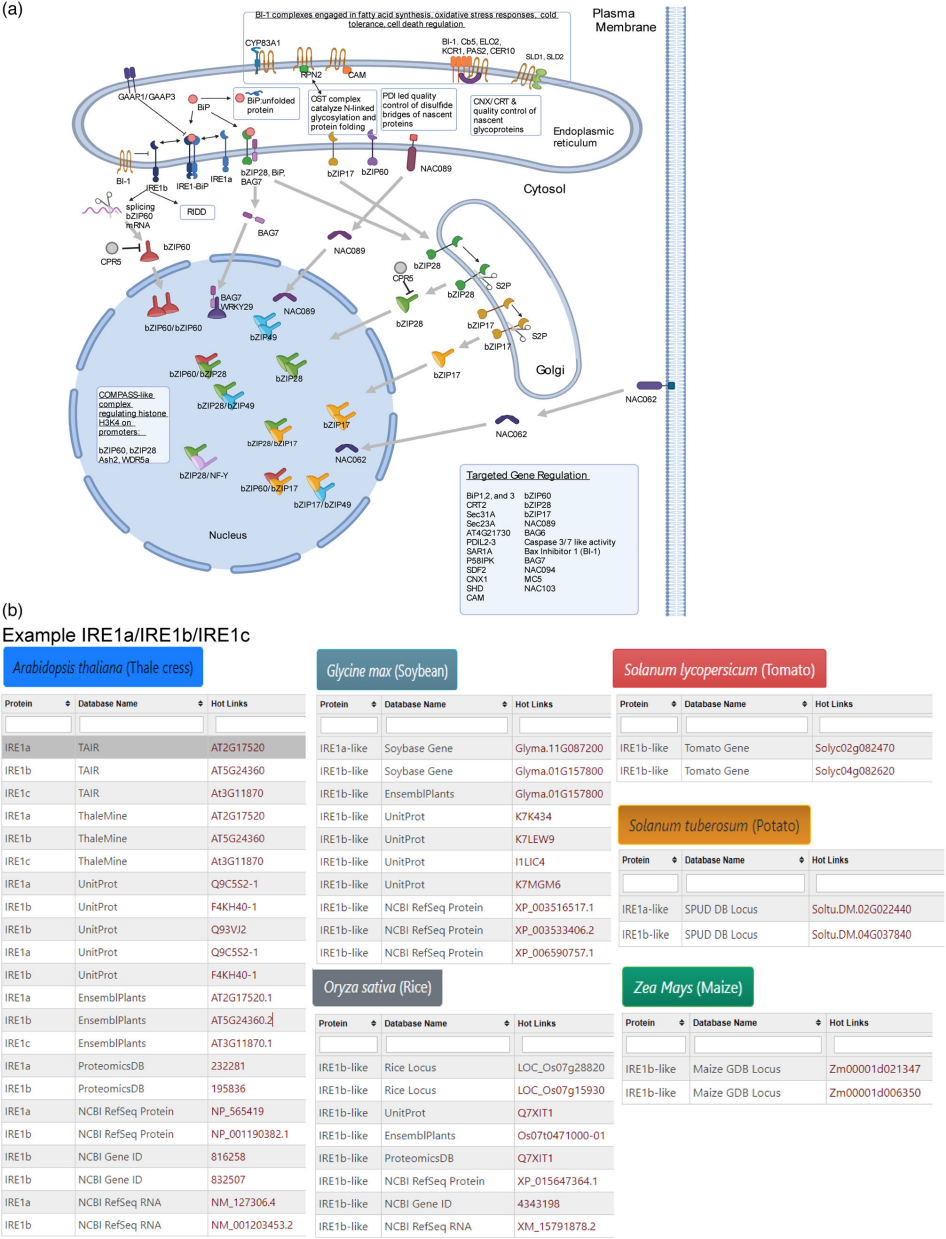
Interactive viewer. (a) A screenshot of the interactive viewer. All the unfolded protein response (UPR) components are hotlinked so the users can obtain their information by clicking on each component. (b) A use case illustrating the retrieval of IRE1 information. When users click on IRE1a or IRE1b, it will open the relevant gene information pages. Users can select one of the six species by clicking on the species name logo. It will bring weblinks for major databases.

The Plant ER UPRome database includes an interactive diagram that shows factors associated with the ER UPR (Figure 3a). The users can click on one protein in the schematic, and then several tabs will appear representing each of six plant species. The next step is to select a plant species, and then a drop‐down list of databases and corresponding accessions will appear (Figure 3b). These accessions are hotlinks that take you to the associated database where users can obtain more in‐depth information about the gene or protein including the gene model, locus information, sequence, subcellular location, tissue expression patterns, and protein structure. For example, by clicking on IRE1a or IRE1b in the diagram (based on Arabidopsis as the primary model reference genome), a bar appears for IRE1a/b/c. Since little is known about the subcellular location of IRE1c, it is not featured in the diagram, but readers are provided information of its existence in Arabidopsis. The Arabidopsis tab provides the locus accessions in TAIR at the top of the list, and all other database accessions follow. The IRE1a, IRE1b, and IRe1c orthologues in the first column deliver direct access to additional information about these genes, though more plant species have two genes identified as IRE1a and IRE1b or as IRE1a‐like or IRE1b‐like. IRE1c has not yet been identified in other plants. While viewing information under the potato, maize, rice, tomato, and soybean tabs, there can be more than one gene entry with the same name. In these cases, such common name can be attributed to these genes in the database but not confirmed experimentally. Two good examples are the maize and rice genomes which have two loci identified as IRE1b‐like. Such ambiguities point out opportunities for further investigations to clarify functional orthologues through more research efforts. One more example is bZIP17. Single loci under the Arabidopsis, potato, maize, tomato, and soybean tabs represents bZIP17. Because no accessions occurs under the rice tab, a note appears saying “External Identifiers‐NOT AVAILABLE for selected plant species.” Upon selecting the potato tab and then the Spud DB locus accession, the hotlink directs you to the Spud DB Potato Genomics Resource page for the locus. This gene in the SPUD DB is identified as a bZIP transcription factor family protein, however, Herath and Verchot (2021) identified this gene as *StbZIP17*; the putative ortholog of Arabidopsis *bZIP17*. This locus is also identified in NCBI as *StbZIP17*. After clicking on BiP in the diagram, a bar appears at the bottom indicating that there are three BiP genes named *BiP1*, *BiP2*, and *BiP3*. Upon selecting Arabidopsis, there appears three primary loci in TAIR and 12 total database accessions. When selecting potato, only three accessions for the SPUD DB loci appear because there are no corresponding accessions in other databases. The rice genome supposedly encodes two *BiP* genes, although the public databases present eight NCBI and EMBL accession identifiers. Similarly, the tomato genome database indicates that there are four *BiP* genes but UniProt shows only one accession. There are no other entries for tomato BiPs in other databases. Therefore to provide accessible and reliable information, the genes in our database that are listed across each plant species has been validated by manually reviewing the literature and information available across all public databases. Thus, we reliably generated specific lists for each species. After clicking on multiple hotlinks in the list, the user can independently validate the gene ID.

The interactive diagram also contains a shaded box entitled “Targeted Gene Regulation”; listing genes whose promoters can be influenced by these signaling machinery. Some promoter targets are confirmed by qRT‐PCR studies, promoter analyses, and transcriptomics studies.

## 4 DISCUSSION

The majority of knowledge of the UPR and ER‐to‐nuclear signaling has been determined based on both forward and reverse genetics studies carried out using Arabidopsis. Advancing efforts to characterize functional orthologues in agronomic hosts has been slow due to many limitations. While there is expanding research on the UPR in agronomically valued crops, many available databases do not fully integrate the available genomic resources across various databases. Identifying conserved factors across plant genome databases can be at times consuming, especially if different genome assemblies show differences in the number of functional orthologues (such as IRE1a/b/c). We launched the Plant ER UPRome project to provide easy access to information concerning ER‐to‐nuclear signaling networks, primarily the UPR, that occur across plant species. We expect the UPR is only a portion of the entire story of ER to nuclear signaling and that a broader repertoire of signaling may occur across plant species, tissue types, or developmental stages. The current UPRome is a high‐quality curated compendium of the fundamental UPR processes in plants. We will strive to expand the compendium to include more plant species and genes as more experimentally validated data are published. The UPRome is an open‐source project shares a visualization of the biological pathways and lists the current and past publications on the topic.

The Plant ER UPRome will be updated routinely by incorporating novel UPR‐associated genes and upcoming literature. We will expand this database to include data from more plant genomes, especially crop genomes. We welcome contributions from the researchers studying the ER and UPR to incorporate additional genes into the database through the “contact us” page where scientists in the community can communicate new information for updating the database. We plan to add new features for functional, structural, and comparative omics studies.

## CONFLICT OF INTEREST

There is no conflict of interest regarding this research.
